# Dissecting microbial community structure and methane-producing pathways of a full-scale anaerobic reactor digesting activated sludge from wastewater treatment by metagenomic sequencing

**DOI:** 10.1186/s12934-015-0218-4

**Published:** 2015-03-14

**Authors:** Jianhua Guo, Yongzhen Peng, Bing-Jie Ni, Xiaoyu Han, Lu Fan, Zhiguo Yuan

**Affiliations:** Key Laboratory of Beijing for Water Quality Science and Water Environmental Recovery Engineering, Engineering Research Center of Beijing, Beijing University of Technology, Beijing, 100124 Peoples’ Republic of China; Advanced Water Management Centre (AWMC), The University of Queensland, St Lucia, Brisbane, QLD 4072 Australia; Beijing Drainage Group Co., Ltd, Beijing, 100022 Peoples’ Republic of China

**Keywords:** Waste activated sludge, Anaerobic digestion, Metagenomic sequencing, Microbial community, Methanogenesis pathway, Biological wastewater treatment

## Abstract

**Background:**

Anaerobic digestion has been widely applied to treat the waste activated sludge from biological wastewater treatment and produce methane for biofuel, which has been one of the most efficient solutions to both energy crisis and environmental pollution challenges. Anaerobic digestion sludge contains highly complex microbial communities, which play crucial roles in sludge treatment. However, traditional approaches based on 16S rRNA amplification or fluorescent *in situ* hybridization cannot completely reveal the whole microbial community structure due to the extremely high complexity of the involved communities. In this sense, the next-generation high-throughput sequencing provides a powerful tool for dissecting microbial community structure and methane-producing pathways in anaerobic digestion.

**Results:**

In this work, the metagenomic sequencing was used to characterize microbial community structure of the anaerobic digestion sludge from a full-scale municipal wastewater treatment plant. Over 3.0 gigabases of metagenomic sequence data were generated with the Illumina HiSeq 2000 platform. Taxonomic analysis by MG-RAST server indicated that overall bacteria were dominant (~93%) whereas a considerable abundance of archaea (~6%) were also detected in the anaerobic digestion sludge. The most abundant bacterial populations were found to be *Proteobacteria*, *Firmicutes*, *Bacteroidetes*, and *Actinobacteria*. Key microorganisms and related pathways involved in methanogenesis were further revealed. The dominant proliferation of *Methanosaeta* and *Methanosarcina*, together with the functional affiliation of enzymes-encoding genes (acetate kinase (AckA), phosphate acetyltransferase (PTA), and acetyl-CoA synthetase (ACSS)), suggested that the acetoclastic methanogenesis is the dominant methanogenesis pathway in the full-scale anaerobic digester.

**Conclusions:**

In short, the metagenomic sequencing study of this work successfully dissected the detail microbial community structure and the dominated methane-producing pathways of a full-scale anaerobic digester. The knowledge garnered would facilitate to develop more efficient full-scale anaerobic digestion systems to achieve high-rate waste sludge treatment and methane production.

**Electronic supplementary material:**

The online version of this article (doi:10.1186/s12934-015-0218-4) contains supplementary material, which is available to authorized users.

## Background

Activated sludge process is the most widely used biological wastewater treatment technology. During its over 100-years development, many novel and modified processes have been proposed in order to efficiently meet the more and more stringent discharge and emission limits [1,2]. However, substantial amounts of excess sludge are generated during wastewater treatment, which require further treatment. This accounts for around 60% of the total operational costs of the overall wastewater treatment plant (WWTP) [3]. As one of the most efficient solutions to both energy crisis and environmental pollution challenges, anaerobic digestion is widely applied to reduce the amount of excess sludge, eliminate pathogens and produce methane [4]. In general, the anaerobic digestion process can convert about 40 ~ 60% of the organic solids (excess sludge) to methane (CH_4_), which is a highly valuable hydrocarbon biofuel, generating 36.5 MJ/m^3^ in combustion [5].

Anaerobic digestion sludge contains highly complex microbial communities, which play critical roles in excess sludge treatment, in particular determining the sludge reduction performance and the methane production efficiency. Many molecular methods, such as denaturing gradient gel electrophoresis (DGGE), fluorescent *in situ* hybridization (FISH), 16S rRNA gene and other marker gene low-throughput sequencing, have been previously applied to investigate the microbial community structure in anaerobic systems [6]. However, these low-throughput approaches are not able to completely reveal the detailed microbial community structure due to the extremely complex communities and overwhelming genetic diversities in anaerobic digestion sludge, especially for those low abundant populations though playing important role in the system. Moreover, the approaches based on clone library sequencing of the 16S rRNA gene for ecological investigations of functional microorganisms may result in an overestimation or underestimation of both their numbers and the diversity due to their inherent bias of amplification [7,8].

High-throughput sequencing methods, such as Illumina sequencing and 454 pyrosequencing technologies, have been recently applied as novel and promising methods to characterize the phylogenetic composition and functional potential of complex community [9,10]. So far, several metagenomic studies have been conducted on microbial community analysis in anaerobic digesters using 454 pyrosequencing [11-13]. Compared to 454 pyrosequencing, Illumina sequencing offers significantly greater throughput and is a more cost-effective approach to study the complex environmental microorganisms [14,15]. To date, Illumina sequencing has been applied in several studies with complex microbial communities, such as soil [16], ocean [17], human gut microbes [18] and activated sludge [19,20]. However, so far, little effort has been dedicated to using Illumina sequencing to analyze in detail the microbial community structure including the rare members of the community as well as their functions in anaerobic digesters [21]. In addition, the dominated methane-producing pathway in full-scale anaerobic digesters for treating excess sludge is still unclear.

The aim of this study was to characterize the metagenomic community composition and reveal functional traits in a typical full-scale anaerobic digester. Toward this end, we extracted DNA from a full-scale anaerobic digestion sludge, and conducted high-throughout (around 3.0 gigabases) metagenomic sequencing on the Illumina HiSeq 2000 platform. The microbial community structures, functional profiles, and metabolic pathways of the anaerobic digestion sludge were revealed. In particular, key microorganisms involved in hydrolysis, acidogenesis, acetogenesis and methane production were comprehensively analyzed based on the obtained metagenomics data. Furthermore, the possible genes associated with methanogenesis pathways were highlighted. This study provides insights into the dominant functions of microbes in full-scale anaerobic digesters, thereby facilitating the development of more efficient full-scale systems to achieve a high-rate sludge reduction and methane production.

## Results and discussion

### Operational performance of the full-scale anaerobic digester

This full-scale anaerobic digester was fed with excess activated sludge (around 900 m^3^ per day) with water content of approximately 96%. The temperature was kept around 35°C, i.e. a typical mesophilic digestion process. The detailed operational conditions and the performance of the full-scale anaerobic digester are summarized in Additional file 1: Table S1 (SI). During the sampling period, the anaerobic digester demonstrated a good performance in terms of volatile solids destruction (51% on the average), nutrient balance, and pathogen destruction (above 90%). The volatile fatty acids (VFAs) in the effluent of the digester were significantly low (lower than 800 mg/L), indicating that the anaerobic digestion system was functioning efficiently in converting VFAs to biogas (methane). The daily methane (CH_4_) production rate was around 1500 m^3^/d. The average methane composition accounted for about 70.8% in the biogas.

### Microbial compositions in anaerobic digester

**Figure 1:**
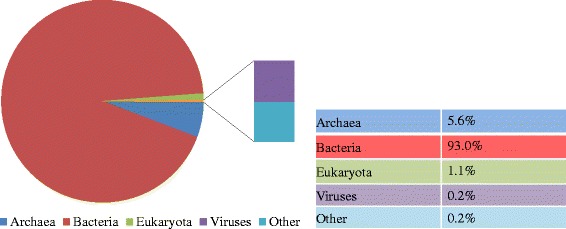
**Taxonomic profiling at the Domain level of the studied anaerobic digestion sludge.** Total DNA sequences were assigned to Bacteria, Eukaryota, Archaea, Viruses, and other sequences.

Overall, Illumina sequencing yielded above 3.0 Gb reads. After quality filtering, the anaerobic digestion sludge yielded more than 2.6 Gb high quality reads. To reveal microbial composition, taxonomic annotation was conducted by Best Hit classification at the E-value cutoff of 10^−5^ with minimum alignment length of 50 bp [21] based on the entire available source databases in MG-RAST. Figure 1 shows that *Bacteria* were the dominant domain in the sample, accounting for 93.0% of anaerobic digestion sludge DNA sequences. Moreover, the abundance of *Archaea* in the anaerobic digestion sludge (5.6%) was slightly higher than those of previous studies [12,21], i.e., below 4.7% of their reads were assigned to *Archaea*. Sequences from *Eukaryota* and *Viruses* only accounted for 1.1% and 0.2% in the anaerobic digestion sludge, respectively. For details, the interactive Krona chart of the full taxonomy can be found in Additional file 1: Figure S1.

**Figure 2:**
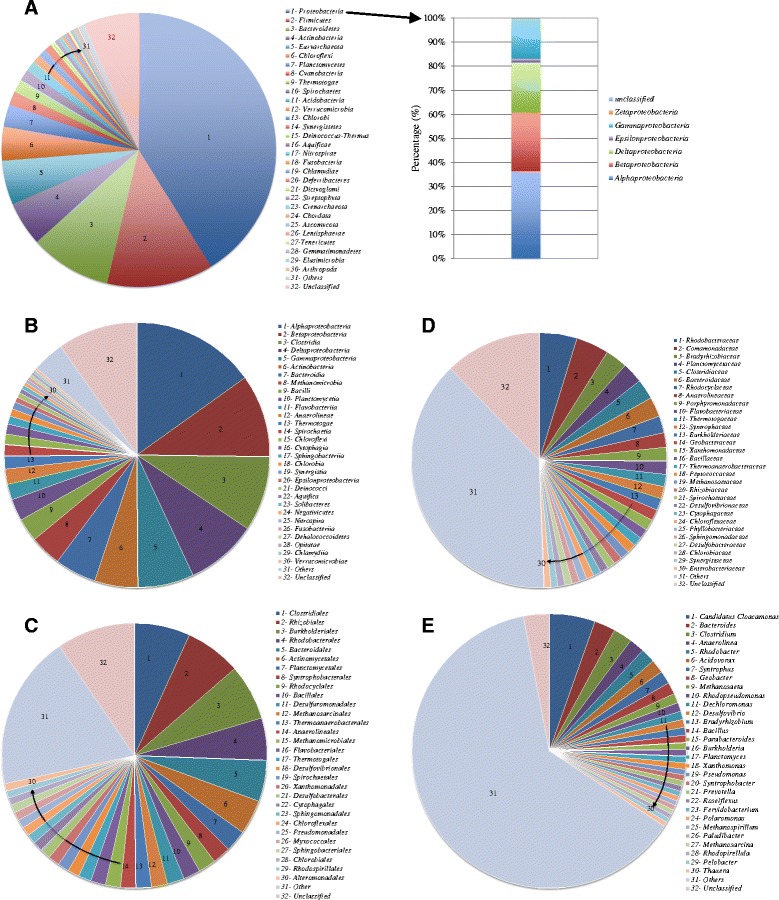
**Pie and bar charts showing taxonomic assignments at the various levels for anaerobic digestion sludge based on metagenomic sequencing data (A: Phylum; B: Class; C: Order; D: Family; E: Genus).**

For a better understanding of the microbial community structure in anaerobic digestion sludge, taxonomic affiliation at different levels was analyzed (Figure 2). At the phylum level, the most abundant bacterial populations were found to be *Proteobacteria*, *Firmicutes*, *Bacteroidetes*, and *Actinobacteria*, accounting for 41.2%, 12.5%, 9.6%, and 5.2% of all the *Bacteria* reads, respectively. *Proteobacteria* are important microbes in anaerobic digestion process because most of *Alpha*-, *Beta*-, *Gamma*-, and *Deltaproteobacteria* are well-known the glucose, propionate, butyrate, and acetate-utilizing microbial communities [22]. At the most abundant phylum *Proteobacteria*, *Alphaproteobacteria* was identified as the most dominant class, having 36.4% of all the classified *Proteobacteria* reads.

Most of members belonging to the *Firmicutes* phylum are syntrophic bacteria that can degrade various VFAs, which were often detected in both activated sludge systems and anaerobic digesters [23]. Within the phylum of *Firmicutes*, *Clostridia* (72.5% of all the *Firmicutes* sequences) and *Bacilli* (22.6%) form the majority of the classes (Additional file 1: Table S3). The class of *Clostridia is* well-known in fermenters. The predominance of *Clostridia* in the anaerobic digestion sludge was associated with a high-rate of hydrolysis and VFA fermentation occurred in the anaerobic digester studied, which was confirmed by the reactor performance data (Additional file 1: Table S1). The genera *Streptococcus* and *Halothermothrix* belonging to the phylum of *Firmicutes* also showed a high abundance based on the metagenomics data (Figure 2).

The major classes within the phylum of *Bacteroidetes* were found to be *Bacteroidia*, *Cytophagia*, *Flavobacteriia* and *Shingobacteriia* (Additional file 1: Table S2). The percentage of *Bacteroidia* was distinctly higher than those of other classes. Similar with the *Clostridia* class, the *Bacteroidaceae* family belonging to *Bacteroidetes* (class) is also well-known comprise fermentative bacteria, which generally play the important role in hydrolyzing and fermenting organic materials and producing organic acids, CO_2_ and H_2_ during the anaerobic digestion process [24].

*Methanomicrobia* were the major class in the phylum of *Euryarchaeota*, taking 85.4% of all the *Euryarchaeota* sequences in the anaerobic digestion sludge (Additional file 1: Table S2). The predominance of *Methanomicrobia* is associated with the abundant methanogens in the sample, in which abundant *Methanosaeta* and *Methanosarcina* are detected (further discussed below).

At the genus level, there are over 2900 different taxa that can be classified (Figure 2). These data demonstrate the extraordinary microbial diversity of anaerobic digestion sludge. The top 50 representing abundant genera in the sample were selected, as shown in Additional file 1: Table S3 (SI). Ten genera have the percentages higher than 1% in the anaerobic digestion sludge. At the genus level, Candidatus *Cloacamonas* is the most dominant taxon in the anaerobic digestion sludge. As reported in previous work [25], Candidatus *Cloacamonas acidaminovorans* is probably a hydrogen-producing syntrophic bacterium that is widely present in many anaerobic digesters.

Recently, the microbial diversity in full-scale biogas production reactors has been reported using metagenomics sequencing [13,21,26]. The current study showed that *Proteobacteria* was the most dominant phylum, followed by *Firmicutes*, *Bacteroidetes*, and *Actinobacteria*, which are consistent with a previous study [21], in which microbial community structure of two full-scale anaerobic digesters operated in municipal WWTPs were revealed through llumina high-throughput sequencing. However through using 454 pyrosequencing of 16S rRNA gene sequences, Sundberg et al. [13] found that the dominant populations included the phyla *Firmicutes*, *Bacteroidetes*, *Actinobacteria*, *Proteobacteria*, *Chloroflexi* and *Spirochaetes* in biogas production reactors digesting sewage sludge, while *Firmicutes* were the most prevalent in codigesting various combinations of wastes from restaurants, households and slaughterhouses. Similarly, a meta-analysis of all publicly available 16S rRNA gene sequences from microbial communities of anaerobic digesters fed with a variety of feedstocks demonstrated that many dominant populations belong to the phyla *Chloroflexi* and *Proteobacteria* [26]. Li et al. [12] also conducted metagenomic analysis of a solid-state biogas reactor based on 647 Mb of data from 454 pyrosequencing. Their results revealed that the most prevalent fermentative microbes are derived from *Clostridiales* (*Firmicutes*). These various dominant populations might be associated with different influent characteristics and operational conditions, which have been reported to strongly influence microbial community structure [13,27-29]. At the WWTP studied in this work, a fraction of industrial wastewater (taking account about 10-20% of the total inflow) was fed into the activated sludge process, subsequently changing the characteristics of the sludge that was fed into the anaerobic digester.

### Global gene functional profiles

**Figure 3:**
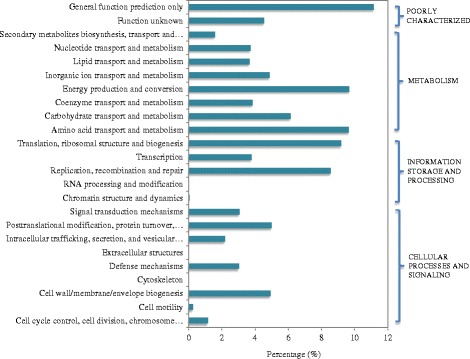
**Functional categories of anaerobic digestion sludge in COG categories.** Name of subcategories in COG database are listed on the left, and the corresponding major categories are list on the right.

To reveal the functional profiling of the full-scale anaerobic digestion sludge, the total reads were annotated according to categories of the COG and KEGG databases (Figure 3 and Additional file 1: Figure S2). COG annotation analysis showed that 43.2% of the total reads were related to metabolism and 19.6% of them were assigned to cellular processes and signaling, whereas about 21.6% corresponded to housekeeping genes involved in information-related processes in anaerobic digestion sludge (Figure 3 and Additional file 1: Table S3). The obtained results are comparable with a previous study [12], in which approximately 28% of the total reads were assigned to one or more COG functional categories and a large number of reads were associated with the metabolism. In the category of metabolism, the most abundant metabolic type was energy production and conversion (9.7%), followed by amino acid transport and metabolism (9.6%) as well as carbohydrate transport and metabolism (6.1%). These metabolic activities are well linked with the conversion of excess activated sludge into methane during anaerobic digestion [21].

The genes involved in amino acid metabolism were detected in reads assigned to “valine, leucine and isoleucine biosynthesis (11695 reads)”, “glycine, serine and threonine metabolism (11027 reads)”, and “cysteine and methionine metabolism (6460 reads)”, which are the three most dominant groups. These amino acids including valine, leucine, isoleucine, glycine, serine, threonine, cysteine and methionine are known to be commonly involved in Stickland reactions. There are principally two pathways in which amino acids can be fermented: (1) pairs of amino acids can be fermented through the Stickland reaction; and (2) single amino acids can be degraded in a process that requires the cooperation with hydrogen-utilizing bacteria [30]. Moreover, the above taxonomic assignment indicated that *Clostridiales* are the first predominant at the order level. Considering that the Stickland reaction has only been reported previously with *Clostridial* species [30], thus, it is most likely that the first pathway, i.e. Stickland reaction, is the predominant amino acid fermentation pathway in this full-scale anaerobic digester. Many of the enzymes involved in the amino acid degradation, such as S-adenosylmethionine synthetase [EC:2.5.1.6], cystine reductase [EC:1.8.1.6], cysteine synthase A [EC:2.5.1.47], alpha-aminoadipic semialdehyde synthase [EC:1.5.1.8; 1.5.1.9] and lysine 2,3-aminomutase [EC:5.4.3.2] are annotated with high reads numbers. These observations are indeed associated with good acidogenesis performance in the anaerobic digester (Additional file 1: Table S1).

There are also abundant reads matching the genes for “carbohydrate metabolism”, mainly including “glycolysis/gluconeogenesis (6,198 reads)”, “pentose phosphate pathway (4918 reads)”, “amino sugar and nucleotide sugar metabolisms (4,525 reads)”, “citrate cycle (TCA cycle, 4149 reads)”, “fructose and mannose metabolism (3223 reads)” and “starch and sucrose metabolism (3141 reads)”, as shown in Figure 3. These annotation observations further confirmed the findings that abundant species in this full-scale anaerobic digester are involved in carbohydrate digestion and energy conversion [21].

**Figure 4:**
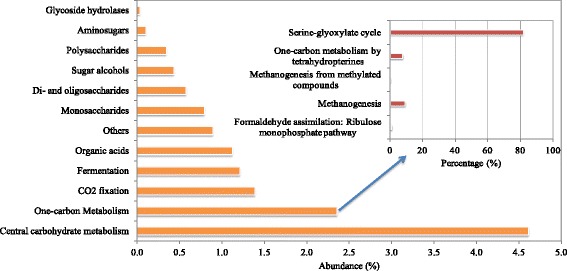
**Abundances of major Level 2 subsystems in anaerobic digestion sludge derived from Level 1 subsystem of carbohydrate based on SEED subsystems (The E-value cutoff of 10** ^**−5**^ **and minimum alignment length of 17 aa was used as the annotation parameters).**

Based on the annotation of functional genes using SEED subsystems in MG-RAST, it was found that the subsystem of carbohydrates was the most abundant one, followed by protein metabolism, amino acids and derivatives (Additional file 1: Table S4). The Level 2 subsystem of carbohydrate was further analyzed and compared. As shown in Figure 4, central carbohydrate metabolism and one-carbon metabolism are the major function in Level 2 subsystems. Central carbohydrate metabolism is used to describe the integration of pathways of transporting and oxidation of main carbon sources inside the cell [31]. It involves a complex series of enzymatic steps to convert external substrate (e.g., sugars) into metabolic precursors, such as acetyl-CoA, pyruvate and d-fructose-6-phosphate [32]. These precursors are then utilized to generate the cell biomass [32]. One-carbon metabolism was also annotated with a high abundance, accounting for 2.33% of the identified carbohydrate subsystem in anaerobic digestion sludge. One-carbon metabolisms convert complex organic matter to simple one-carbon compounds, which play important roles in the process of methanogenesis and are generally present in methanogenic Archaea [33].

### Key microorganisms involved in anaerobic digestion process

**Figure 5:**
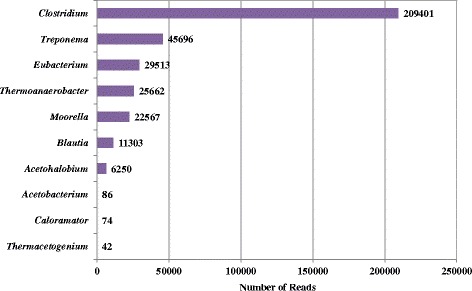
**Key genera of acetogenic bacteria detected in the full-scale anaerobic digester.**

The anaerobic digestion process generally consists the four stages, i.e. hydrolysis, fermentation, acetogenesis and methanogenesis [34]. Various microorganisms are involved in each step and cooperated with each other to achieve high-rate sludge reduction and methane formation in anaerobic digestion. According to the sequencing data of this work, the members of the order of *Halanaerobiales* (mainly the genus *Halothermothrix*) dominated in the anaerobic digester, which are predicted to hydrolyze polymers to monomers with their enzymes, converting particulate materials into dissolved materials at the first stage. Subsequently, the fermentative bacteria, mainly the *Clostridia* class and the *Bacteroidaceae* family in this study, performed the acidogenic process at the second stage and produced VFA, CO_2_ and H_2._ At the third stage, acetogenic bacteria further converted these products to acetate by utilizing obligate hydrogen-producing acetogens or homoacetogens via the pathway of CO_2_ reduction with the acetyl-CoA synthase as the key enzyme. In fact, there are more than 20 bacterial genera that contain over 100 reported acetogenic species in literature [35]. Our results suggests that *Clostridium*, *Treponema*, *Eubacterium*, *Thermoanaerobacter* and *Moorella* are the dominant acetogenic bacteria in the studied anaerobic digester as shown in Figure 5, consistent with a previous reports [29].

**Figure 6:**
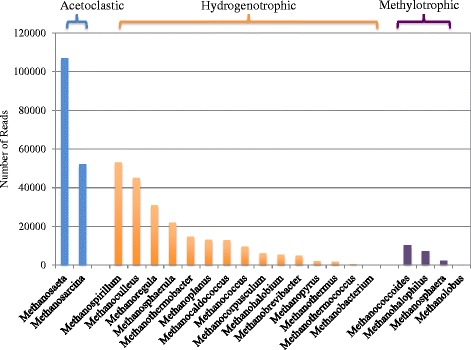
**Key genera involved in methanogenesis process.**

Biological methane production is the last step of anaerobic digestion, in which methanogens are the key microorganisms producing methane as the end product. Methanogens include a phylogenetically diverse group belonging to the *Archaea*. These methanogens are classified into five well-established orders: *Methanobacteriales*, *Methanococcales*, *Methanomicrobiales*, *Methanosarcinales*, and *Methanopyrales*. In this study, key genera involved in the methanogenesis pathway were further analysed based on the obtained sequencing data (Figure 6). The results indicated that the five most dominant methanogenic genera are *Methanosaeta* (26.2% of all the methanogens), *Methanospirillum* (13.1%), *Methanosarcina* (12.8%), *Methanoculleus* (11.1%) and *Methanoregula* (7.6%) in the full-scale anaerobic digestion sludge. Among them, only two genera are known to use acetate for methanogenesis, i.e. *Methanosaeta* and *Methanosarcina. Methanosaeta* is a specialist that uses acetate exclusively, whereas *Methanosarcina* is a relative generalist that can utilize methanol, methylamine and acetate, as well as hydrogen and carbon dioxide for methane production [36]. Hydrogenotrophic methanogens can reduce CO_2_ to CH_4_ with H_2_ as the primary electron donor, as well as formate. A diverse group of hydrogenotrophic methanogens (e.g. *Methanospirillum*, *Methanoculleus* and *Methanoregula*) was detected in the full-scale anaerobic digester; however, their abundances were lower than acetoclastic methanogens. Moreover, the methylotrophic methanogens such as *Methanococcoides, Methanohalophilus* and *Methanolobus* are also found with a relatively lower reads number in the anaerobic digestion sludge. In addition, a number of *Methanosphaera* belonging to the order *Methanobacteriales* were also found in the anaerobic digestion sludge, which are able to use methanol.

### Dissecting the pathways involved in methanogenesis

**Figure 7:**
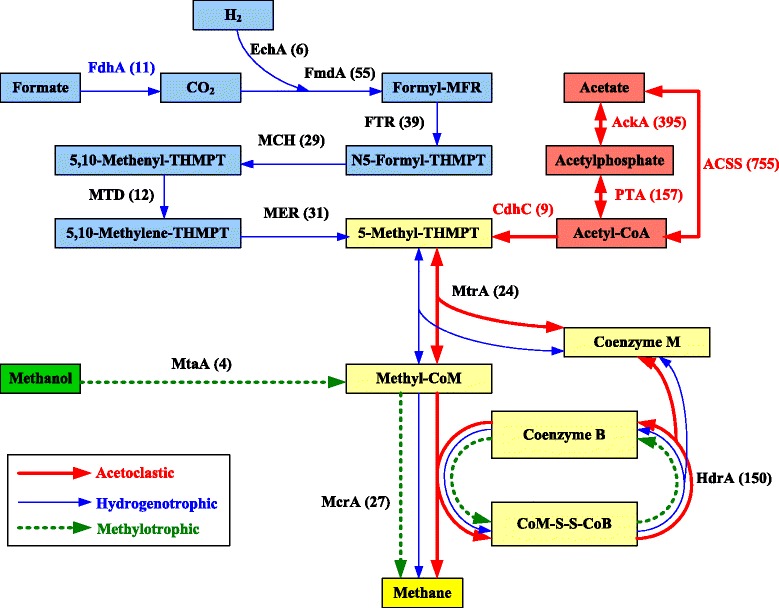
**Hits numbers of genes involved in the relevant methanogenesis pathways in anaerobic digestion.** The three known pathways involved in methanogenesis are colored differentially. The acetoclastic pathway is shown in red, the hydrogenotrophic pathway is marked in green, and the methylotrophic pathway is presented in green. FdhA, glutathione-independent formaldehyde dehydrogenase; EchA, hydrogenase subunit A; FmdA, formylmethanofuran dehydrogenase subunit A; FTR, formylmethanofuran-tetrahydromethanopterin N-formyltransferase; MCH, methenyltetrahydromethanopterin cyclohydrolase; MTD, methylenetetrahydromethanopterin dehydrogenase; MER, coenzyme F420-dependent N5, N10-methenyltetrahydromethanopterin reductase; MtrA, tetrahydromethanopterin S-methyltransferase; MtaA, [methyl-Co(III) methanol-specific corrinoid protein]:coenzyme M methyltransferase; McrA, methyl-coenzyme M reductase alpha subunit; AckA, acetate kinase; ACSS, acetyl-CoA synthetase; PTA, phosphate acetyltransferase; HdrA, heterodisulfide reductase subunit A; CdhC, acetyl-CoA decarbonylase/synthase complex subunit beta.

In order to reveal the dominant methanogenesis pathway, functional enzyme-encoding genes for the relevant methanogenesis pathways in the anaerobic digester were identified and annotated with reference to a methanogenesis genes database extracted from KEGG (Figure 7). According to current knowledge, there are mainly three recognized methanogenic pathways, including acetoclastic, hydrogenotrophic and methylotrophic pathways [36]. For hydrogenotrophic methanogenesis, CO_2_ is reduced successively to CH_4_ through a series of intermediates, including formyl, methylene, and methyl levels. The methyl group is then transferred to Coenzyme M, forming methyl-CoM. The methyl-CoM is reduced to CH_4_ through methyl coenzyme M reductase (Mcr) at the final step (blue line in Figure 7). For the acetoclastic pathway, acetate is firstly converted to acetyl-CoA, in which *Methanosarcina* utilizes the low-affinity acetate kinase (AK)-phosphotransacetylase (PTA) system to activate acetate to acetyl-CoA, while *Methanosaeta* uses the high-affinity adenosine monophosphate (AMP)-forming acetyl-CoA synthetase. Then acetyl-CoA is converted to a methyl group and subsequently to methane through the key enzymes of Cdh, Mtr and Mcr (red line in Figure 7). For methylotrophic pathway, the methyl-groups from methylated compounds or methane are transferred to a methanol-specific corrinoid protein (green dashed line in Figure 7). Methyl-CoM subsequently enters the methanogenesis pathway and is then reduced to methane via Mcr reductase.

Based on the obtained results, the abundances of genes encoding enzymes in acetoclastic pathway are much higher than that involved in hydrogenotrophic and methylotrophic pathways. For instance, the abundances of AckA and PTA are 395 and 157 hits, respectively, while the abundances of FmdA and FTR are 55 and 39 hits, respectively. Compared to the genes of the hydrogenotrophic pathway, the abundances of genes in methylotrophic pathway were the lowest among the three methanogenesis pathways. The obtained results suggested that acetoclastic pathway is likely the major pathway of methane production in anaerobic digestion processes [21,37]. However, it should be noted that the abundance of genes in methanogenesis pathway was based on metagenomics (DNA level), rather than metatranscriptomics or metaproteomics (RNA or protein level), which are required to further explore the active functions involved in the methanogenesis pathway in future study.

## Methods

### Sampling of full-scale anaerobic digestion sludge

The anaerobic digestion sludge was collected from the anaerobic digester from a full-scale WWTP, Beijing, China. This WWTP treats a mean influent flow of 1,000,000 m^3^/day and services a population of approximately 2,400,000 people in Beijing. The excess sludge from the biological treatment process is removed via the secondary clarifiers and enters the sludge treatment units together with the primary sludge. The sludge treatment processes consists of thickening tanks, anaerobic mesophilic digestion and dewatering. The process diagram and the detailed operational condition are shown in Additional file 1: Figure S3 and Table S1 (Supporting information, SI), respectively. Samples were mixed with 100% ethanol at a ratio of 1:1 (volume/volume) immediately after being collected from the full-scale anaerobic digester, then transferred to the lab using an ice-box and stored at −20°C before the DNA extraction.

### DNA extraction

Genomic DNA extraction was conducted within 24 hours after sampling. Around 2 mL sample was centrifuged at 3750 g for 5 min to collect the sludge pellet by removing the supernatant. DNA extractions were performed using the FastDNA SPIN Kit for Soil (QBIOgene Inc., Carlsbad, CA, USA), according to the manufacturer’s instructions. DNA quality was assessed using gel electrophoresis (1% agarose) and DNA concentrations were determined using a Qubit Fluorometer (Thermo, USA). The DNA concentration of anaerobic digestion sludge was 580 ng/*μ*L.

### DNA library construction and sequencing

The metagenomic sequencing was conducted using Illumina HiSeq 2000 platform by the Beijing Genomic Institute at Shenzhen, China. The extracted DNA sample was afterwards processed according to the genomic DNA sample preparation kit protocol (Illumina). The DNA fragmentation was firstly performed using Covaris S2 Ultrasonicator. The DNA fragments were then processed by end reparation, A-tailing, adapter ligation, DNA size-selection, PCR reaction and products purification based on Illumina HiSeq 2000 instructions. For sequencing, a library consisting of approximate 170 bp fragments was constructed. The base-calling pipeline (version Illumina Pipeline-0.3) was used to process the raw fluorescence images and call sequences. The sequencing depth of 3.0 Gb reads was applied for the sample metagenomic datasets. The metagenomic reads were trimmed using a minimum quality score of 30, a minimum read length of 35 bp and allowing no ambiguous nucleotides. The parameters adopted for overlapping were as follows: at least 20 nt length of the overlap region was required, and at most two mismatches were allowed.

### Bioinformatic analyses

Unassembled DNA sequences were annotated using the Metagenomics Rapid Annotation (MG-RAST) server (v3.1). MG-RAST not only enables phylogenetic and metabolic reconstructions, but also provides protein similarities analysis, including both function annotation and function classification [38]. In the present study, 3.0 Gbp DNA dataset (MG-RAST ID: 4536159.3) was used for most of the analysis. Taxonomic profiles were calculated by Best Hit classification at the E-value cutoff of 10^−5^ with minimum alignment length of 50 bp based on all the annotation source databases used by MG-RAST. The distribution of taxonomic domains, phyla, orders, families and genus for the annotations was analysed in detail. Concerning taxonomic profiles, percentages shown in the study referred to those classified at a certain taxonomic level.

Functional profiling was conducted by the gene annotation with SEED Subsystems using Hierarchical classification at E-value cutoff of 10^−5^ and minimum alignment length of 17 amino acids [21,39], respectively, in MG-RAST, and visualized using KEGG mapper. Most of the genes were successfully classified into the hierarchical metabolic categories.

To investigate gene profile characteristic for the anaerobic microbial community, the total sequencing reads were annotated against the databases of Clusters of Orthologous Groups of proteins (COG) and Kyoto Encyclopedia of Genes and Genomes (KEGG, v59) [40,41] databases using BLASTP (v2.2.21) with the E-value cutoff of 10^−5^. Detailed analysis of the anaerobic digestion sludge was conducted to count and compare the hit numbers of the sequences of corresponding enzymes subunits in the methanogenesis pathways. The module ‘KEGGviewer’ in MEGAN was used to analyze pathways [42,43]. Proteins glutathione-independent formaldehyde dehydrogenase (FdhA), hydrogenase subunit A(EchA), formylmethanofuran dehydrogenase subunit A (FmdA), formylmethanofuran-tetrahydromethanopterin N-formyltransferase (FTR), methenyltetrahydromethanopterin cyclohydrolase (MCH), methylenetetrahydromethanopterin dehydrogenase (MTD), coenzyme F420-dependent N5, N10-methenyltetrahydromethanopterin reductase (MER), tetrahydromethanopterin S-methyltransferase (MtrA), [methyl-Co(III) methanol-specific corrinoid protein]:coenzyme M methyltransferase (MtaA), methyl-coenzyme M reductase alpha subunit (McrA), acetate kinase (AckA), acetyl-CoA synthetase (ACSS), phosphate acetyltransferase (PTA), heterodisulfide reductase subunit A (HdrA), acetyl-CoA decarbonylase/synthase complex subunit beta (CdhC) play important roles in recognized methanogenesis pathways, but lack good representative sequences in the eggNOG and KEGG databases at the time of this study. To accurately discover them, BLASTX results were manually analysed through keyword searches based on NCBI-nr annotations, in which genes representing top BLASTX matches were recovered from GenBank. Confirmation of methanogenesis genes was conducted by manually aligning the matched sequences against NCBI-nr database (9 June 2014) using BLAST with E-value cutoff of 10^−10^.

## Conclusions

This study successfully dissected the detailed microbial community structure and the key methane-producing pathways of a full-scale anaerobic digester through applying metagenomics approach. Taxonomic analysis indicated *Proteobacteria*, *Firmicutes*, *Bacteroidetes*, and *Actinobacteria* are the four most abundant bacterial populations in anaerobic digestion sludge. For full-scale anaerobic digester treating sewage sludge, the production of methane is achieved through consortia of microorganisms (hydrolysers, fermenters, acetogens and methanogens) working in a step-wise reaction. The members of the order of *Halanaerobiales* (mainly the genus *Halothermothrix*) are the major hydrolysers, while the *Clostridia* class and the *Bacteroidaceae* family are the dominant fermenters in the system. *Clostridium*, *Treponema*, *Eubacterium*, *Thermoanaerobacter* and *Moorella* are found to play important roles on acetate production at the acetogenesis step. The dominant proliferation of the acetoclastic methanogens (*Methanosaeta* and *Methanosarcina*), together with the functional affiliation of enzymes-encoding genes (Ack, PTA, ACSS, etc.), strongly suggested that the acetoclastic methanogenesis might be the dominant methanogenesis pathway in the anaerobic digester. Further studies directly based on metatranscriptomics or metaproteomics are necessary to further explore the active functions in the full-scale biogas production digester.

## Additional file

Additional file 1: Table S1. Operation condition and performance of the second-stage anaerobic digester in a full-scale WWTP. **Table S2.** Percentage of dominant class in major phylum from Bacteria and Archaea. **Table S3.** Abundances of Top 50 genera in the ADS sample. The abundance is presented in terms of percentages of the total sequences in the sample. **Table S4.** Level 1 subsystems in the ADS sample, annotated by SEED sub-systems databases with E-value cutoff of 1e-5 and minimum alignment length of 17 aa. **Figure S5.** The interactive Krona chart of the full taxonomy. **Figure S6.** KEGG mapper for the anaerobic digestion sample. **Figure S7.** Schematic diagram of the full-scale wastewater treatment plant and the sampling point (sampling point as shown by a red star).
