# Supplementary material for: Dissecting microbial community structure and methane-producing pathways of a full-scale anaerobic reactor digesting activated sludge from wastewater treatment by metagenomic sequencing
Source: Microb Cell Fact. 2015 Mar 14;14:33. doi: 10.1186/s12934-015-0218-4 (PMC4381419; doi:10.1186/s12934-015-0218-4)
Supplement: Additional file 1: Table S1. — Operation condition and performance of the second-stage anaerobic digester in a full-scale WWTP. Table S2. Percentage of dominant class in major phylum from Bacteria and Archaea. Table S3. Abundances of Top 50 genera in the ADS sample. The abundance is presented in terms of percentages of the total sequences in the sample. Table S4. Level 1 subsystems in the ADS sample, annotated by SEED sub-systems databases with E-value cutoff of 1e-5 and minimum alignment length of 17 aa. Figure S5. The interactive Krona chart of the full taxonomy. Figure S6. KEGG mapper for the anaerobic digestion sample. Figure S7. Schematic diagram of the full-scale wastewater treatment plant and the sampling point (sampling point as shown by a red star). [file 12934_2015_218_MOESM1_ESM.docx]

**Supplementary Materials for**

**Dissecting microbial community structure and methane-producing** **pathways through metagenomic sequencing in a full-scale anaerobic digester**

Jianhua Guo^1,2*^, Yongzhen Peng^1^, Bing-Jie Ni^2^, Xiaoyu Han^1^, Lu Fan^2^, Zhiguo Yuan^2^

^1^ Key Laboratory of Beijing for Water Quality Science and Water Environmental Recovery Engineering, Engineering Research Center of Beijing, Beijing University of Technology, Beijing 100124, PR China

^2^ Advanced Water Management Centre (AWMC), The University of Queensland, St Lucia, Brisbane, QLD 4072, Australia

*Corresponding authors: Jianhua Guo, Phone: +61 (0)7 3346 3215; Fax: +61 (0)7 3365 4726;

E-mail: [gjh@bjut.edu.cn](mailto:gjh@bjut.edu.cn); [j.guo@awmc.uq.edu.au](mailto:j.guo@awmc.uq.edu.au)

**List of tables**

Table S1. Operation condition and performance of the second-stage anaerobic digester in a full-scale WWTP

**Table S2**. Percentage of dominant class in major phylum from Bacteria and Archaea

**Table S3**. Abundances of Top 50 genera in the ADS sample. The abundance is presented in terms of percentages of the total sequences in the sample

**Table S4**. Level 1 subsystems in the ADS sample, annotated by SEED sub-systems databases with E-value cutoff of 1e-5 and minimum alignment length of 17 aa

**List of figure**

**Figure S1.** The interactive Krona chart of the full taxonomy

**Figure S2**. KEGG mapper for the anaerobic digestion sample

**Figure S3**. Schematic diagram of the full-scale wastewater treatment plant and the sampling point (sampling point as shown by a red star)

**Table S1** Operation condition and performance of the second-stage anaerobic digester in Gaobeidian WWTP

|  | Volume  (m^3^) | Temperature  (^o^C) | pH | Retention time  (d) | Sludge feeding volume  (m^3^) | VS  (%) | H_2_S  (%) | CH_4_  (%) | CO_2_  (%) |
| --- | --- | --- | --- | --- | --- | --- | --- | --- | --- |
| Range | ­­-- | -- | 7.3-7.8 | -- | -- | 22-87 | 0.1-0.4 | 53.8-74.2 | 17.6-30.8 |
| Average | 7080 | 35 | -- | 5 | 900 | 51 | 0.3 | 70.8 | 22.8 |
| STD | -- | -- | -- | -- |  | 15 | 0.1 | 3.0 | 2.5 |
| No. | -- | -- | -- | -- | -- | 63 | 54 | 55 | 55 |

**Table S2**. Percentage of dominant class in major phylum from Bacteria and Archaea

| Phylum | Class | Abundance percentage (%) |
| --- | --- | --- |
| *Proteobacteria* | *Betaproteobacteria* | 24.32 |
|  | *Deltaproteobacteria* | 20.94 |
|  | *Alphaproteobacteria* | 36.43 |
|  | *Gammaproteobacteria* | 16.47 |
|  | *Epsilonproteobacteria* | 1.50 |
|  | *Others* | 0.35 |
| *Firmicutes* | *Clostridia* | 72.50 |
|  | *Bacilli* | 22.56 |
|  | *Erysipelotrichi* | 1.62 |
|  | *Negativicutes* | 3.32 |
| *Bacteroidetes* | *Bacteroidia* | 52.44 |
|  | *Cytophagia* | 12.54 |
|  | *Flavobacteriia* | 19.76 |
|  | *Sphingobacteriia* | 10.25 |
|  | *Others* | 5.01 |
| *Euryarchaeota* | *Methanomicrobia* | 85.41 |
|  | *Thermoplasmata* | 1.13 |
|  | *Methanobacteria* | 6.04 |
|  | *Halobacteria* | 2.98 |
|  | Others | 4.44 |

**Table S3**. Abundances of Top 50 genera in the ADS sample. The abundance is presented in terms of percentages of the total sequences in the sample

| **ADS** | **ADS** | **ADS** |
| --- | --- | --- |
| **Phylum** | **Genus** | **Percentage (%)** |
| *unclassified Bacteria* | *Candidatus* Cloacamonas | 5.40 |
| *Bacteroidetes* | *Bacteroides* | 2.48 |
| *Firmicutes* | *Clostridium* | 2.22 |
| *Chloroflexi* | *Anaerolinea* | 1.83 |
| *Proteobacteria* | *Rhodobacter* | 1.70 |
| *Proteobacteria* | *Acidovorax* | 1.59 |
| *Proteobacteria* | *Syntrophus* | 1.53 |
| *Proteobacteria* | *Geobacter* | 1.40 |
| *Euryarchaeota* | *Methanosaeta* | 1.13 |
| *Proteobacteria* | *Rhodopseudomonas* | 1.04 |
| *Proteobacteria* | *Dechloromonas* | 0.97 |
| *Proteobacteria* | *Desulfovibrio* | 0.94 |
| *Proteobacteria* | *Bradyrhizobium* | 0.92 |
| *Firmicutes* | *Bacillus* | 0.87 |
| *Bacteroidetes* | *Parabacteroides* | 0.81 |
| *Proteobacteria* | *Burkholderia* | 0.79 |
| *Planctomycetes* | *Planctomyces* | 0.78 |
| *Proteobacteria* | *Xanthomonas* | 0.75 |
| *Proteobacteria* | *Pseudomonas* | 0.66 |
| *Proteobacteria* | *Syntrophobacter* | 0.66 |
| *Bacteroidetes* | *Prevotella* | 0.64 |
| *Chloroflexi* | *Roseiflexus* | 0.63 |
| *Thermotogae* | *Fervidobacterium* | 0.61 |
| *Proteobacteria* | *Polaromonas* | 0.58 |
| *Euryarchaeota* | *Methanospirillum* | 0.56 |
| *Bacteroidetes* | *Paludibacter* | 0.55 |
| *Euryarchaeota* | *Methanosarcina* | 0.55 |
| *Planctomycetes* | *Rhodopirellula* | 0.55 |
| *Proteobacteria* | *Pelobacter* | 0.54 |
| *Proteobacteria* | *Thauera* | 0.52 |
| *Proteobacteria* | *Ruegeria* | 0.51 |
| *Actinobacteria* | *Streptomyces* | 0.49 |
| *Acidobacteria* | *Candidatus* Solibacter | 0.49 |
| *Planctomycetes* | *Pirellula* | 0.49 |
| *Spirochaetes* | *Treponema* | 0.48 |
| *Euryarchaeota* | *Methanoculleus* | 0.48 |
| *Proteobacteria* | *Mesorhizobium* | 0.47 |
| *Planctomycetes* | *Blastopirellula* | 0.47 |
| *Spirochaetes* | *Spirochaeta* | 0.47 |
| *Proteobacteria* | *Rhizobium* | 0.45 |
| *Bacteroidetes* | *Chlorobium* | 0.45 |
| *Proteobacteria* | *Nitrobacter* | 0.44 |
| *Thermotogae* | *Thermotoga* | 0.44 |
| *Bacteroidetes* | *Pedobacter* | 0.42 |
| *Proteobacteria* | *Roseobacter* | 0.41 |
| *Proteobacteria* | *Sinorhizobium* | 0.41 |
| *Firmicutes* | *Desulfotomaculum* | 0.41 |
| *Actinobacteria* | *Mycobacterium* | 0.41 |
| *Proteobacteria* | *Paracoccus* | 0.40 |
| *Proteobacteria* | *Caulobacter* | 0.40 |

**Table S4**. Level 1 subsystems in the ADS sample, annotated by SEED sub-systems databases with E-value cutoff of 1e-5 and minimum alignment length of 17 aa

| **Level 1** | **Abundance/%** |
| --- | --- |
| Amino Acids and Derivatives | 8.53 |
| Carbohydrates | 9.95 |
| Cell Division and Cell Cycle | 1.57 |
| Cell Wall and Capsule | 3.32 |
| Clustering-based subsystems | 15.16 |
| Cofactors, Vitamins, Prosthetic Groups, Pigments | 6.12 |
| DNA Metabolism | 5.34 |
| Dormancy and Sporulation | 0.24 |
| Fatty Acids, Lipids, and Isoprenoids | 2.52 |
| Iron acquisition and metabolism | 0.45 |
| Membrane Transport | 3.11 |
| Metabolism of Aromatic Compounds | 1.14 |
| Miscellaneous | 7.77 |
| Motility and Chemotaxis | 0.73 |
| Nitrogen Metabolism | 1.18 |
| Nucleosides and Nucleotides | 3.32 |
| Phages, Prophages, Transposable elements, Plasmids | 1.96 |
| Phosphorus Metabolism | 0.82 |
| Photosynthesis | 0.06 |
| Potassium metabolism | 0.33 |
| Protein Metabolism | 10.71 |
| RNA Metabolism | 4.45 |
| Regulation and Cell signaling | 1.20 |
| Respiration | 3.46 |
| Secondary Metabolism | 0.38 |
| Stress Response | 2.49 |
| Sulfur Metabolism | 0.85 |
| Virulence, Disease and Defense | 2.83 |

**
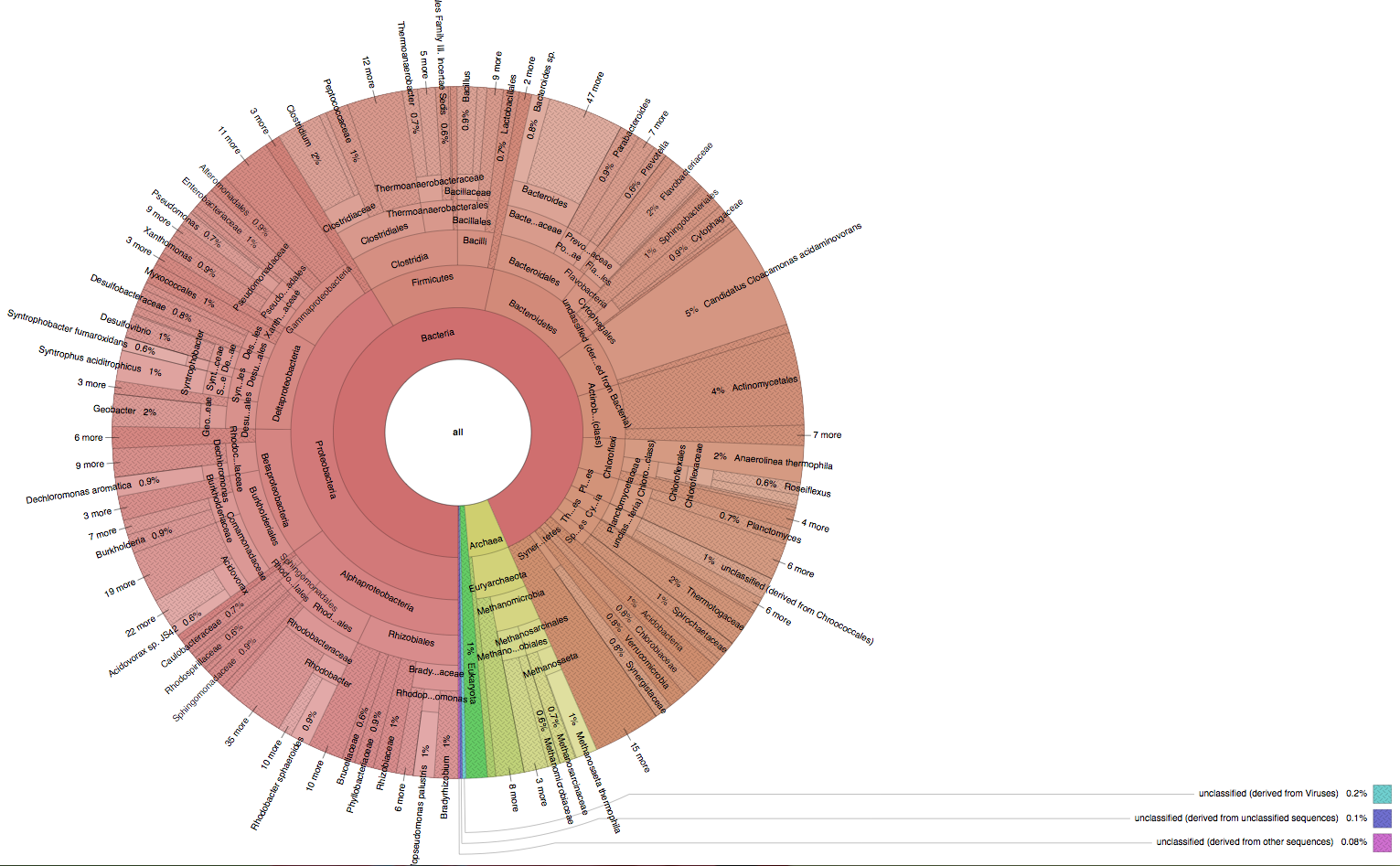
**

**Figure S1.** The interactive Krona chart of the full taxonomy

**
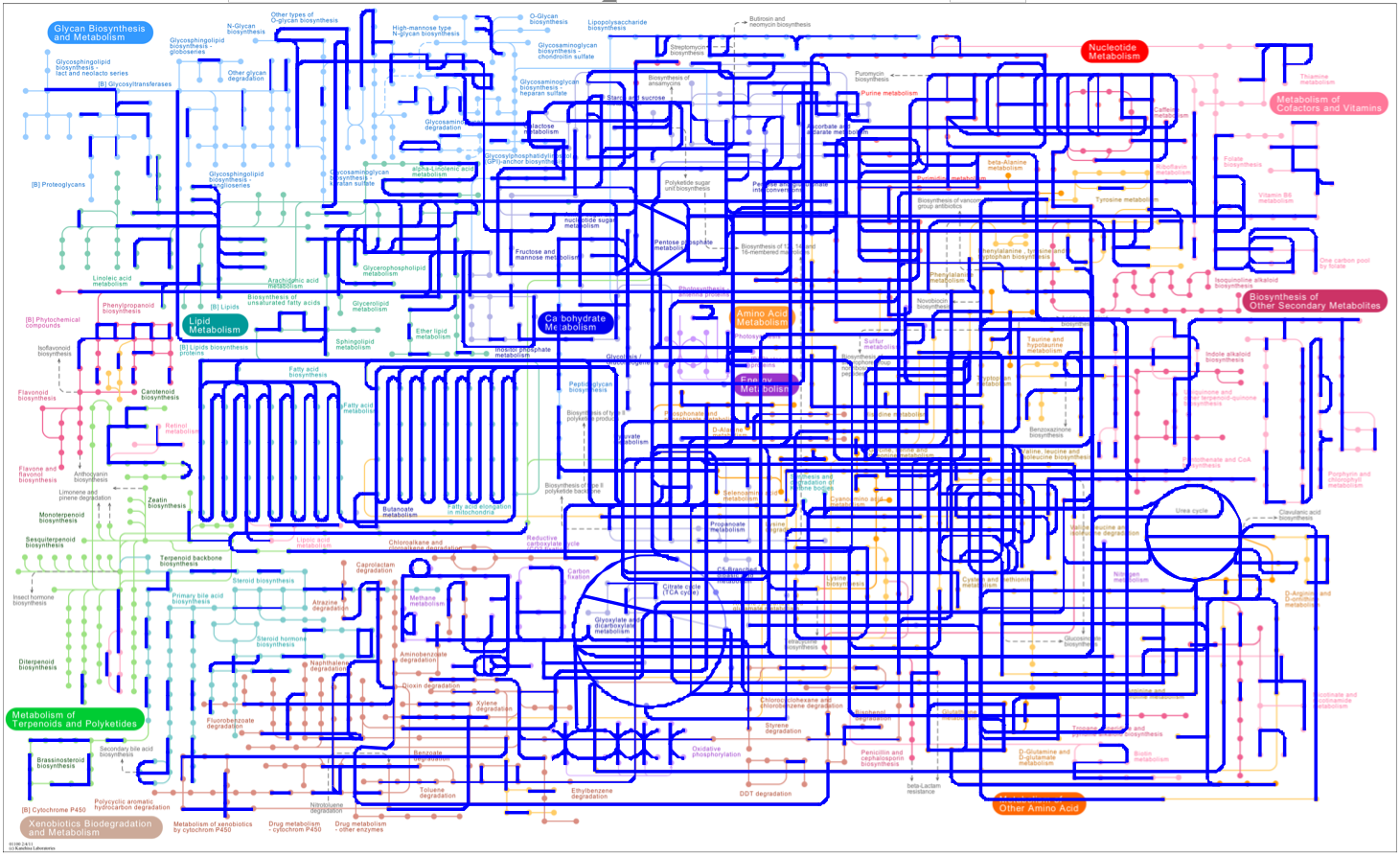
**

**Figure S2.** KEGG mapper for the anaerobic digestion sample


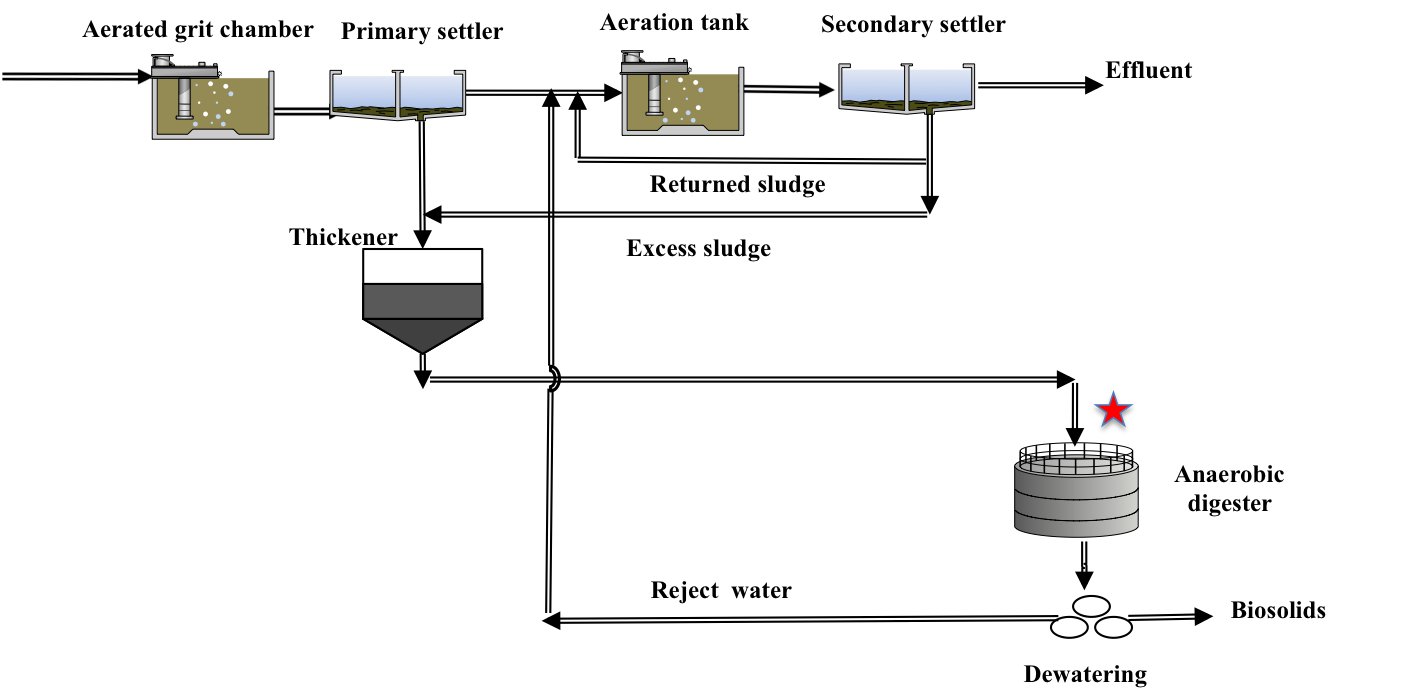


**Figure S3**. Schematic diagram of the full-scale wastewater treatment plant and the sampling point (sampling point as shown by a red star)
